# Determinants of speeding among new generations of car drivers from the Arabian Peninsula. An investigation based among Omani drivers using the theory of planned behaviour

**DOI:** 10.1371/journal.pone.0226441

**Published:** 2019-12-16

**Authors:** Constance Boissin, Abdullah Ali Al Maniri, Ali Sulieman Al-Azri, Marie Hasselberg, Lucie Laflamme

**Affiliations:** 1 Department of Global Public Health, Karolinska Institute, Stockholm, Sweden; 2 Studies and Planning Department, Oman Medical Specialty Board, Muscat, Oman; 3 Directorate of Planning, Ministry of Health, Muscat, Oman; Tsinghua University, CHINA

## Abstract

In high-income countries of the Arabian Peninsula, including the Sultanate of Oman, motorization has been extremely rapid. As a result, road traffic crashes are by far the highest cause of premature mortality, and speeding is an acknowledged key risk factor. Theory-based interventions are needed to target prevention of this unsafe practice. This study sheds light on determinants of speeding among new generations of Omani drivers applying the Theory of Planned Behaviour (TPB). A questionnaire covering all five main constructs of the TPB was first contextualized and administered to two target groups: male drivers of all ages (n = 1107) approached in person when renewing their driving license and university students drivers (men and women) reached through internet contact (n = 655). Multiple, stepwise linear regression analyses were used to explore factors associated with speeding. Results indicate that driving fast and not respecting the posted speed limits was common in both groups of drivers, although rates were higher among students; 41.8% reported driving a bit faster than other drivers and 24.1% faster than the posted speed limit compared with 31.4% and 14.2% in male drivers of all ages. In both groups the TPB model predicted to a limited extent the determinants of speeding behaviour. However, the intention to speed was associated with a negative attitude towards the respect of rules for men of all ages (β = -0.30 (p<0.001)) and for students (β = -0.26 (p<0.001)); a positive view regarding subjective norms (β = 0.25 (p<0.001) and β = 0.28 (p<0.001) respectively), and behavioural control (β = 0.15 (p<0.001) and β = 0.20 (p<0.001) respectively). Intention was the only significant predictor of speeding behaviour (β = 0.48 (p<0.001); and β = 0.64 (p<0.001)). To conclude, speeding is widespread among Omani drivers of all ages and the intention to respect posted speed limits meets a range of barriers that need greater consideration in order to achieve a better safety culture in the country.

## Introduction

In recent years the road traffic environments of most countries of the Arabian Peninsula have undergone major changes. This includes the emergence of numbers of kilometres of modern road traffic infrastructures, a rapidly growing fleet of private motor vehicles–above all modern and powerful vehicles–and with this, a large number of new generations of motor vehicle drivers, many of whom drive long distances. This has been accompanied by the emergence of high-risk behaviours among motor-vehicle drivers and alarmingly high rates of road traffic injuries, fatal and non-fatal [1–4]. In the Sultanate of Oman alone, the setting of the current study, road traffic crashes (RTC) have become the first cause of premature mortality, representing nearly one third of all deaths (28%) and the country has the world’s second highest rate of fatal RTC [5].

Speeding, defined as “driving faster than the posted speed limit or too fast for the prevailing conditions” [6] is acknowledged as one of the major causes of fatal crashes worldwide. In Oman, studies reveal that about half of RTC are speed-related [7,8]. In an effort to discourage speeding, the Omani authorities have taken important steps such as posting speed limits, speed cameras, vehicle registration, and road worthiness centres [9]. To date however, speeding remains common on the road and the individual factors related to this practice in the population have not been thoroughly investigated. Identifying these factors would help guide the promotion of safe driving as a “voluntary” practice and contribute to the introduction of a safe culture on the road. Behavioral changes of the like may be long-term but are essential as currently the acceptance of speeding is high, the road infrastructure facilitates it, and the geography challenges the application of speed controls and other various forms of law enforcement prevention efforts. Against this background, this study was embarked upon in order to enhance our understanding of the determinants of speeding using the Theory of Planned Behaviour (TPB) as a framework [10]. Two specifically high-risk groups of drivers in the Omani context were targeted: male drivers [7,11,12] and young drivers [11].

The TPB proposes that behaviour is determined by two main individual factors: the intention (or the willingness to behave in a certain manner) and the perceived behavioural control. The intention for its part is determined by three factors: the attitudes towards performing a behaviour (much related to the positive or negative evaluation of the expected output of the behaviour), the social norms (the perceived social pressure in engaging in the behaviour), and the perceived behavioural control (the perceived ability in performing the behaviour). The theory of planned behaviour has been used in speeding studies in very different settings, including the United Kingdom [13–16], Sweden [17–19], Iran [20], Malaysia [21], and Ghana [22]. These studies indicate that the relative importance of each of the constructs in predicting behaviour can depend on context and population groups [19]; for example, while attitudes played an important role in predicting intention to speed among drivers from the United Kingdom and Sweden [15,18], attitudes did not predict intention among Iranian commercial drivers [20]. The need to clarify the individual factors likely to influence speeding behaviour in countries of the Arabic Peninsula has previously been put forward by several researchers from the region [23,24].

We addressed the following research questions targeting both male drivers and young drivers (males and females) who are more prone to speeding:

- What are the determinants of speeding among Omani drivers based on the theory of planned behaviour?
- How well does the theory of planned behaviour contribute to explain speeding among these drivers?

## Materials and methods

Two cross-sectional sub-studies among two groups of Omani drivers were conducted to provide theory-based evidence through questionnaires to understand factors related to their speeding behaviour.

### Participants

*Sub-study group I*. Data collection took place between March 2^nd^ and March 20^th^, 2013 in the governorate of Muscat. The sub-study targeted only male participants. The inclusion criteria included drivers aged 18–45 years who were able to read and write and had a valid motor vehicle driving licence. Participants were recruited while sitting in the waiting room of the Directorate of Vehicle Registration of the Royal Oman Police in the Muscat governorate, Alseeb Wilayate. A systematic, random sampling procedure was employed whereby the man sitting on seat number one was first asked to participate and from then on every third man. If a person did not match the inclusion criteria, the next person was approached and given a questionnaire. Once all those who had accepted to participate had filled in a questionnaire (paper copy), a new round started. A sample size of 1800 was estimated using an expected speeding prevalence of 50% in the population and a response rate of 85%. Out of the 1800 questionnaires distributed, 1286 were answered, obtaining a response rate of 71.4%.

*Sub-study group II*. Data collection took place from April to October 2015 in nine governmental educational institutions using an internet-based and self-administered online survey, accessed using an individual link sent to each participant’s individual email. This sub-study targeted young male and female students approached through their educational institution. We used a stratified cluster sampling technique considering two strata: one of governmental and one of private colleges/universities. In each stratum one representative cluster was selected from each governorate, except for Muscat where two clusters were generated to reflect the higher population size. All the sampling units within the selected cluster (institution) were targeted. However, no private educational institutions were included because of the difficulty in contacting them. Out of the nine governmental educational institutions included, eight confirmed that they had distributed the survey to their students and 966 students completed the survey.

### Data collection instrument

A questionnaire was developed by the authors of this study to assess speed-determinants using the theory of planned behaviour complemented by questions relative to the respondent’s socio-demographic characteristics, individual driving history, practice and habits as a car driver, and own experience of RTC as a car driver. Questions related to driving history included the number of years since obtaining a driving license and the number of kilometres driven in a week. It also included a question asking whether the respondent had been involved in a RTC as a driver. Questions related to driving practice and habits included the respondent’s speeding behaviour compared to other drivers, the occurrence of having had a ticket for speeding, and the frequency of driving faster than or in respect to the posted speed limits in different road environments. The questionnaire was first validated by five experts in the field who reviewed the logical links between the items and the constructs, as well as whether the items covered the subject in breadth. Construct validity and reliability were then assessed using a pilot study including 90 respondents.

Each of the theory’s constructs was built based on a set of questions and items stated hereafter. All items were presented with a five-point Likert-type response scale. To measure *attitudes* towards exceeding the speed limit, seven items were used. As opposed to the other constructs, all items capture a dimension that reveals a negative attitude towards respect of the posted speed limits. These were for example “Exceeding the speed limit every time I drive my car will make me feel like a ‘real man’” or “Exceeding the speed limit every time I drive my car will save my time” (scale from strongly agree to strongly disagree). Four items were used to measure *subjective norms*. These were “I am always persuaded to observe the speed limits by my colleagues at work / my family / my friends / Islamic beliefs” (scale from strongly agree to strongly disagree). To measure *perceived behavioural control* towards the behaviour of respecting the speed limit, five items were used. These were, for example “These issues make it easier for me to respect the speed limits–the feeling that I could be stopped by the police” (scale from strongly agree to strongly disagree). *Behaviour* was also assessed using a self-reported measure in order to increase potential sample size, and considering that a number of studies have shown that there is a relatively high correlation between self-reported and observed speeding behaviour [15,25,26]. *Behaviour* and *intention* were therefore assessed considering four different environments (on highway, main roads, internal streets and service roads) with a question formulated as follows: For *behaviour* (i.e. respecting the speed limit), “In the last months, how often, would you say you have respected the speed limit when driving a car?” (scale from very often / always to never). For *intention* (to respect the speed limit) “In the next month, I intent to respect the speed limit every time that I take my car” (scale from strongly agree to strongly disagree).

The same questionnaire was used for the two different study groups (men of all ages, and university students), however some differences were introduced in the wording of a number of questions and some questions were asked in only one survey (e.g., monthly income and having children in the first survey and gender in the second one).

### Data treatment

Data from the two sub-studies were analysed separately using Stata/SE 15.0 for Mac, and all the data used can be found in S1 Table. We report on the results of the analyses related to the constructs of the TPB by considering only those respondents who answered all related questions: 1107 participants for study group I (men of all ages; 86.1%); and 655 for study group II (students; 67.8%) including respondents for whom the gender question remained unanswered. Table 1 presents the characteristics of the respondents from both sub-studies.

**Table 1:** Characteristics of the respondents from study groups I and II.

Characteristics	Mean (SD)	N	%
**Study group I–**Men of all ages (n = 1107)			
**Age (in years**)	29.3 (6.6)		
18–20		62	5.6
21–25		325	29.4
26–30		303	27.4
31–35		208	18.8
> = 36		209	18.9
**Education**			
Basic reading and writing		32	2.9
Secondary education or less		459	41.5
First university degree		559	50.5
Post-graduate		57	5.2
**Study group II–**Students (n = 655)			
**Age (years)**	21.1 (1.9)		
18–19		145	22.1
20–22		367	56.0
23–25		143	21.8
**Gender**			
Men		375	57.3
Women		169	25.8
Unknown		111	16.9

For each construct the mean of all items was calculated for each individual to create a composite scale to be used in the subsequent analyses. Cronbach’s alpha was calculated for each construct to assess for reliability of the data. The mean score of each construct on a scale from one to five was used to look at the correlation between each construct and all four others.

To test the extent to which the theory of planned behaviour fitted in each study group, we proceeded to two sets of stepwise multiple linear regressions, one for intention and one for behaviour. For both analyses, in the first step, individual socio-demographic characteristics were introduced into the model. These included for both sub-study groups age, mileage and crash involvement (having been involved in a road traffic crash while driving in the last three years; dichotomized with reference to crash involvement). For study group I, education level (dichotomized, with reference category secondary education or less) was also included. For study group II, gender (expressed as two individual variables: men versus women and unknown; and women versus men and unknown) was added. In the second step, we introduced the theory of planned behaviour’s constructs: when considering how well intention was predicted, attitudes, subjective norms, and behavioural control were introduced in the model; and when considering the prediction of behaviour, intention and behavioural control were introduced.

### Ethical considerations

Ethics approval was obtained from the ethics committee at the Sultan Qaboos University (MREC#651). Written informed consent from the participants was obtained prior to any data collection.

## Results

Table 2 presents information about the driving experiences of the two sub-study groups. Respondents from study group I (men of all ages) drove longer distances in a week in greater proportions than those from study group II (students); they had owned a driving license for a longer time and 36.2% had been involved in a road traffic crash as a driver, in comparison with 31.3% of the students.

**Table 2:** Driving experience of the respondents from study groups I and II.

Characteristics	Study group I–Men of all ages (n = 1107) %	Study group II–Students (n = 655) %
**Total kilometres in a week**		
<100	8.5	27.3
100–300	26.7	32.8
>300	64.8	39.8
**Time since getting a driving license (in years)**		
< 1	0.0	30.2
1–2	10.7	29.5
3–4	13.4	27.5
5+	75.1	12.8
Unanswered	0.8	0.0
**History of having been involved in a RTC as a driver**		
Yes	36.2	31.3
No	63.8	68.7
**Own speeding behaviour compared to other drivers**		
Much slower	1.4	1.2
A little slower	12.6	12.7
About the same	50.0	44.3
A little faster	27.0	36.6
Much faster	4.3	5.2
Unanswered	4.6	0.0
**Having had a ticket for speeding**		
Yes	46.6	40.8
No	53.4	59.2
**D****riving faster** **than the speed limit**		
Never / very rarely	13.4	9.8
Occasionally	41.1	31.8
Sometimes	30.4	34.4
Often	10.6	16.2
Very often / always	3.9	7.9
Unanswered	0.7	0.0
**Respecting the speed limit** **on highways**		
Very often / always	41.6	31.6
Often	38.4	32.1
Occasionally	15.1	24.1
Very rarely	3.4	9.6
Never	1.5	2.6
**Respecting the speed limit** **on main roads**		
Very often / always	47.0	33.7
Often	35.5	39.5
Occasionally	12.7	20.0
Very rarely	3.5	4.7
Never	1.3	2.0
**Respecting the speed limit** **on internal streets**		
Very often/always	57.7	46.6
Often	21.0	25.0
Occasionally	13.3	19.2
Very rarely	5.1	6.4
Never	2.9	2.7
**Respecting the speed limit** **on service roads**		
Very often/always	56.4	41.5
Often	21.9	31.6
Occasionally	12.3	18.6
Very rarely	6.1	5.8
Never	3.3	2.4

When comparing themselves to other drivers, a very small proportion of respondents from both groups reported that they drove much slower than other drivers (1.4% and 1.2% respectively) and the majority reported that they drove about the same or a bit faster than others (50.0% and 27.0% for men of all ages; 44.3% and 36.6% for students respectively). Most respondents from both groups reported driving faster than the speed limits, either occasionally or sometimes. However, about 25% of the university students (compared to 15% in the all-ages group) reported driving faster than the limit often (16.2% compared with 10.6%) or very often/always (7.9% compared with 3.9%). Among the students, similar proportions of males and females reported driving faster than the speed limit often or very often (26.1% and 24.3% respectively), and both of those proportions were higher than the ones reported by the (men) drivers from study I (18.5% for those 25 years or younger and 12.4%, for the older ones). In each study, reporting adherence to the speed limit very often/always was more prevalent on internal streets and service roads than on highways and main roads.

Table 3 presents the items-specific answers of the respondents of each study for the constructs on attitudes and subjective norms, highlighting the proportion of respondents answering that they agreed or strongly agreed with a given statement. The statements are ordered in decreasing order of agreement for all respondents of Study 1 aggregated. For the attitudes concerning how exceeding the speed limits would make one feel, the respondents from Study 1 were less incline to agree/strongly agree with any of the statements than the university students from Study 2. Further, the proportions were consistently higher among the younger than older men (Study 1) and in all instances but one among the female compared to the male university students. Statements of note among female students were that exceeding the speed limit will “increase their driving skills” (40.8%), “save their time” (39.6%) and “increase their ability to overtake” (36.1%). Regarding subjective norms, overall all respondents were in high agreement with the statements related to the influence of both Islamic thoughts (90.2% and 83.4% for respondents in Study I and II respectively) and of their family (90.1% and 87.8% for participants in Study I and II respectively). For students, the latter ranked higher than the former, especially for women (91.7% for their family compared to 84.0% for Islamic thoughts). The influence of work colleagues was overall much lower, and even more so for students of Study II (31.5% agreed/strongly agreed compared to 57.8% of the respondents from Study I).

**Table 3:** Percentage of respondents’ who agreed or strongly agreed with each of the individual items included in the constructs on attitudes and subjective norms, by age for study group I and by gender for study group II.

	Study group I–Men of all ages			Study group II–Students		
		Age			Gender	
*Attitudes*	All N = 1107	18–25 years N = 387	>25 years N = 720	All N = 655^a^	Men N = 375	Women N = 169
Exceeding the speed limit every time I drive my car will…	%	%	%	%	%	%
Give the impression that I am brave	8.0	11.9	5.8	14.1	12.5	20.7
Make me feel like I am a “real man”	8.1	10.6	6.8	8.1	6.9	8.3
Make me feel like I trust my driving skills	13.5	18.9	10.6	27.2	27.5	32.0
Give the impression that my car is a good car	15.1	22.2	11.3	23.5	25.8	23.1
Increase my ability to overtake	16.2	22.7	12.6	30.8	31.7	36.1
Increase my driving skills	16.4	24.3	12.1	36.3	36.0	40.8
Save my time	17.6	24.6	13.9	31.0	29.1	39.6
*Subjective norms*						
Respecting the speed limits is persuaded to me always by…						
Islamic thoughts	90.2	87.6	91.5	83.4	82.1	84.0
My family	90.1	88.6	90.8	87.8	86.4	91.7
My friends	71.2	68.2	72.8	53.9	47.5	62.7
My colleagues at work	57.8	61.2	56.0	31.5	26.4	34.9

^a^111 of the students had unknown gender

Table 4 presents the internal properties of the constructs of the TPB for each study group. There was good internal reliability for all constructs, with Cronbach’s alpha values ranging from 0.780 to 0.981 in the all-ages group and from 0.766 to 0.920 in the student group. In both groups attitudes had the strongest internal reliability.

**Table 4:** Characteristics of the constructs included in the theory of planned behaviour for study groups I and II.

Construct	No. of items	Cronbach’s α	Mean	SD
Study group I–Men of all ages (n = 1107)				
1. Behaviour	4	0.831	1.79	0.81
2. Behavioural intention	4	0.900	1.59	0.75
3. Attitudes	7	0.931	4.02	0.97
4. Subjective norms	4	0.780	1.86	0.77
5. Perceived behavioural control	5	0.814	2.46	1.02
Study group II–Students (n = 655)				
1. Behaviour	4	0.782	2.03	0.80
2. Behavioural intention	4	0.883	1.99	0.86
3. Attitudes	7	0.920	3.50	1.01
4. Subjective norms	4	0.766	2.19	0.78
5. Perceived behavioural control	5	0.863	2.34	1.03

Table 5 shows how each construct was correlated to one another. In the all-ages group, all constructs were significantly correlated to one another (i.e., correlation coefficients were significant; p<0.05) and in the student group, all but attitudes with subjective norms.

**Table 5:** Correlations between the constructs included in the theory of planned behaviour for study groups I and II.

Construct	1	2	3	4	5
Study group I–Men of all ages (n = 1107)					
1. Behaviour	–	0.504**	-0.281**	0.195**	0.113**
2. Behavioural intention		–	-0.327**	0.291**	0.168**
3. Attitudes			–	-0.103**	0.046
4. Subjective norms				–	0.139**
5. Perceived behavioural control					–
Study group II–Students (n = 655)					
1. Behaviour	–	0.659**	-0.212**	0.351**	0.131**
2. Behavioural intention		–	-0.287**	0.332**	0.238**
3. Attitudes			–	0.003	0.089*
4. Subjective norms				–	0.196**
5. Perceived behavioural control					–

* Correlation coefficients p<0.05

** Correlation coefficients p<0.01

Common to both study groups was the fact that behaviour was more strongly correlated to intention than to perceived behavioural control. The highest correlation coefficient in both groups was, by far, between (speeding) behaviour and behavioural intention (0.504 and 0.659 respectively). Furthermore, behavioural intention was most strongly correlated to negative attitudes in the all-ages group, and to the subjective norms in the student group. Also, in both groups the correlations were negative between attitudes to exceeding the speed limit with both behavioural intention and behaviour to respect the speed limit.

Table 6 shows the stepwise linear regression models predicting the intention (i.e. not to respect the speed limit) for each study group. In both studies the individual socio-demographic characteristics as a whole accounted for a very small, but significant proportion of the variance (R^2^ = 0.02 and R^2^ = 0.04 for men of all ages and students respectively). Age was negatively associated with intention in both groups, whereas previous crash involvement was significantly associated with intention in the all-ages group only. When adding the TPB constructs into the model at step 2, there was a significant increase in the proportion of variance in intention explained by the model (R^2^_change_ = 0.18 for men of all ages and R^2^_change_ = 0.21 for students). Then, educational level in the all-ages group was the only personal characteristic that was individually associated with intention, and none of the respondents’ characteristics remained associated in the students’ group. Furthermore, all three constructs included in the theory of planned behaviour as potential predictors (attitudes towards exceeding the speed limit, subjective norms and perceived behavioural control) were strongly associated with intention. Attitudes had the strongest prediction and was negatively associated with intention (β = -0.30 in the all-ages group and β = -0.26 in the student group), whereas subjective norms (β = 0.25 and β = 0.28 in the all-ages and student groups respectively) and perceived behavioural control (β = 0.15 and β = 0.20 in the two groups respectively) provided a positive prediction.

**Table 6:** Association between behavioural intention of not respecting the speed limit and theory of planned behaviour’s constructs using stepwise linear regression for study groups I and II.

				*F*_*change*_		Step 1		Step 2	
Predictors	Step	R^2^	R^2^_change_	Value	P value	β	P value	β	P value
Study group I–Men of all ages (n = 1107)									
1.	Age	0.02	0.02	5.73	<0.001	-0.12	<0.001	-0.04	0.144
	Education level					0.00	0.883	-0.05	0.046
	Mileage					0.03	0.311	0.01	0.721
	Crash involvement					0.06	0.047	0.04	0.159
2.	Attitudes	0.20	0.18	83.49	<0.001			-0.30	<0.001
	Subjective norms							0.25	<0.001
	Perceived behavioural control							0.15	<0.001
Study group II–Students (n = 655)									
1.	Age	0.04	0.04	5.37	<0.001	-0.11	0.007	-0.07	0.065
	Gender (men vs women or missing)					0.13	0.018	0.07	0.163
	Gender (women vs men or missing)					0.05	0.321	0.02	0.624
	Mileage					0.11	0.008	0.06	0.114
	Crash involvement					0.02	0.646	0.00	0.984
2.	Attitudes	0.25	0.21	58.79	<0.001			-0.26	<0.001
	Subjective norms							0.28	<0.001
	Perceived behavioural control							0.20	<0.001

For each study group, Table 7 shows how behaviour (non-respect of the speed limit) was predicted by individual characteristics and by the two constructs of the theory of planned behaviour that were expected to predict it. In both groups the individual socio-demographic characteristics as a whole again accounted for a very small but significant proportion of the variance (R^2^ = 0.04 and R^2^ = 0.07 for men of all ages and students respectively). When adding the theory of planned behaviour’s constructs into the model at step 2 there was a significant increase in the proportion of variance in behaviour explained by the model (R^2^_change_ = 0.24 for men of all ages and R^2^_change_ = 0.39 for students). Then, in both groups, only behavioural intention–not perceived behavioural control–was associated with not respecting the speed limit.

In other words, negative intentions, i.e. a lack of willingness to respect the speed limits, were likely to predict the actual exceeding of those limits, even after controlling for factors like individual socio-demographic characteristics and experience on the road. This does not apply to perceived behavioural control.

**Table 7:** Association between the behaviour of not respecting the speed limit and theory of planned behaviour’s constructs using stepwise linear regression for study groups I and II.

				F_change_		Step 1		Step 2	
Step	Predictors	R^2^	R^2^_change_	Value	P value	β	P value	β	P value
Study group I–Men of all ages (n = 1107)									
1.	Age	0.04	0.04	11.77	<0.001	-0.20	<0.001	-0.14	<0.001
	Education level					-0.02	0.541	-0.02	0.525
	Mileage					-0.03	0.322	-0.05	0.063
	Crash involvement					0.02	0.495	-0.01	0.745
2.	Intention	0.28	0.24	178.41	<0.001			0.48	<0.001
	Perceived behavioural control							0.04	0.141
Study group II–Students (n = 655)									
1.	Age	0.07	0.07	9.80	<0.001	-0.16	<0.001	-0.09	0.004
	Gender (men vs women or missing)					0.18	0.001	0.10	0.017
	Gender (women vs men or missing)					0.11	0.038	0.07	0.069
	Mileage					0.13	0.001	0.07	0.037
	Crash involvement					0.07	0.156	0.04	0.142
2.	Intention	0.46	0.39	228.86	<0.001			0.64	<0.001
	Perceived behavioural control							-0.03	0.270

In sum, in both study groups all three constructs expected to predict the behavioural intention of speeding actually do predict it, but only behavioural intention–and not perceived behavioural control–predicts the behaviour of not respecting the posted speed limits. This, in turn, suggests that behavioural intention can be a crucial target in population-based interventions and all three constructs that predict it deserve close attention.

## Discussion

When asked, many Omani drivers admitted driving faster than the posted speed limit, either often or very often (15% and 25% of the respondents in men drivers of all ages and students respectively). According to the theory of planned behaviour model, in both groups a negative attitude towards respecting the rules, subjective norms and perceived behavioural control were all predictors of the intention to drive faster than the posted speed limits. In fact, together they contributed to explaining about 20% of the variance in intention (20% for men of all ages, 25% in the student group). Also, in both groups the behaviour itself was strongly predicted by intention but not by perceived behavioural control.

In our study 45% of men drivers of all-ages and 58% of students reported driving faster than the speed limit at least sometimes. These high rates, likely to be underestimated due to being self-reported, are in line with those of other studies–although using different measures–from Oman [1] and the Arabic and neighbouring regions like from the United Arab Emirates [24,27], Saudi Arabia [28] and Iran [20]. University students reported both driving fast and not respecting the posted speed limits to a greater extent than the other group, as it has already been previously demonstrated [29]. One unexpected result was that female students while reporting driving faster than the posted speed limit less often, agreed and strongly agreed to a slightly larger extent to the statements on attitudes regarding how speeding would make one feel. How does gender-based differences compare in the region is uncertain due to the paucity of the literature at hand [20,23]. They might be a bit more salient than in neighbouring countries like the United Arab Emirates where both genders answered similarly to items of the Driver Behaviour Questionnaire such as “disregard the speed limits late at night or early in the morning” [27].

As shown in similar previous studies based on the TPB model conducted in the United Kingdom, Sweden and Malaysia [14–16, 18,21], attitudes, subjective norms and perceived behavioural control were all predictors of the intention to speed. Also, the fact that behaviour was associated with only intention in both our sub-study groups was in line with previous studies dealing with either self-reported behaviour [17,18], as in our case, and prospectively observed behaviours [15]. To explain the lack of association between perceived behavioural control and behaviour, it has been suggested that experienced drivers believe that they have control over their driving, whether speeding or not [17]. However, this would hardly contribute to the answers provided by as many as one third of the students whose experience in driving was minimal (less than one year).

An unexpected result was that the constructs of the model explained a large variance in driving behaviour, especially in students (up to 46%), which is much higher than intention (20% and 25% for the two groups respectively). This contrasts with the results of a meta-analysis looking at the prediction of different behaviours which showed that overall the model explains 39% of the variance in intention, but only 27% of the variance in behaviour [30]. It is of note, however, that the variances observed in our study are quite comparable to the ones shown among Iranian drivers [20]. Whether this is an indication of differences between types of countries remains to be determined. This could also find an explanation in the data collection method whereby both intention and behaviour were measured through self-reported rather than observed behaviours.

To our knowledge this is the first study that explores self-reported speeding behaviours using TPB in the Omani–and even more widely in the Arabian Peninsula–context. This is a knowledge gap that has been emphasized in the past [23,24]. Results can be used for the planning of long-term theory-driven public health interventions, meaning that drivers’ attitudes, subjective norms and perceived behavioural control could be targeted in order to change their intention and behaviour with regards to respecting the posted speed limits. There are reasons to believe that the results obtained are robust, as the study includes two large high-risk groups of participants: men of all ages, and university students. Although the first study group was recruited at the Royal Oman Police Directorate of Vehicle Registration, we believe that participants were not strongly influenced as their attendance was not associated with a speeding behaviour. In the second study group the relatively low response rate of the university students is probably due to the electronic aspect of the questionnaire, which made reaching out to the target group challenging.

One limitation of this study is the use of self-reported measures, especially for behaviour, which could lead to potential information bias. However, studies have shown that there is a high correlation between self-reported and observed speeding behaviour [15,25,26], with self-reported behaviour measures being a good proxy for observed speeding in relation to the TPB. Furthermore, this outcome variable was measured in all study groups in the same manner, thus the relative differences observed should not be influenced by this bias. The model can be seen as a useful instrument for public health interventions and the results are in favour of using it to explain speeding behaviours. However, it does not account for extended versions of the TPB which include variables such as anticipated regret, moral norms or past experience and which have been used to explain an even larger proportion of the variance in intention and behaviour [13, 16,26].

## Conclusion

Like in other HICs of the Arabian Peninsula, speeding is highly prevalent among Omani drivers. in this study of young students and drivers of all ages. In accordance with the TPB, the speeding behaviour was predicted by intention. This in turn was predicted by all three constructs of attitudes, subjective norms and perceived behavioural control. Interventions should be tailored to target Omani drivers’ attitudes and subjective norms in order to influence a change in intention to speed. This is key in a setting like that of the Sultanate of Oman, where law enforcement alone is not able to influence the driving culture due to the geography of the country and the incentives found in the road traffic environment itself. More complex, culture-specific interventions are therefore needed. Then, future research will be required to assess both the short and long term impact on driving behaviour changes that will take time to produce effects. This new knowledge may influence the development of such programs in neighbouring countries facing similar challenges.

## Supporting information

S1 Table Dataset of all cases and variables used. (XLSX)
